# *Escherichia coli* cell factories with altered chromosomal replication scenarios exhibit accelerated growth and rapid biomass production

**DOI:** 10.1186/s12934-022-01851-z

**Published:** 2022-06-21

**Authors:** Hee Jin Yang, Kitae Kim, Soon-Kyeong Kwon, Jihyun F. Kim

**Affiliations:** 1 Department of Systems Biology, Division of Life Sciences, Institute for Life Science and Biotechnology, and Microbiome Initiative, Yonsei University, 50 Yonsei-ro, Seodaemun-gu, Seoul, 03722 Republic of Korea; 2 Division of Applied Life Science (BK21), Gyeongsang National University, 501 Jinju-daero, Jinju-si, 52828 Gyeongsangnam-do Republic of Korea; 3 Department of Systems Biology, Yonsei University, 50 Yonsei-ro, Seodaemun-gu, Seoul, 03722 Korea

**Keywords:** Replication profile, Replication strategy, Ectopic replication origins, Multiple replication origins, Cell cycle, Aberrant replication origins, Genomic architecture engineering, Biomass increase

## Abstract

**Background:**

Generally, bacteria have a circular genome with a single replication origin for each replicon, whereas archaea and eukaryotes can have multiple replication origins in a single chromosome. In *Escherichia coli*, bidirectional DNA replication is initiated at the origin of replication (*oriC*) and arrested by the 10 termination sites (*terA*–*J*).

**Results:**

We constructed *E. coli* derivatives with additional or ectopic replication origins, which demonstrate the relationship between DNA replication and cell physiology. The cultures of *E. coli* derivatives with multiple replication origins contained an increased fraction of replicating chromosomes and the cells varied in size. Without the original *oriC*, *E. coli* derivatives with double ectopic replication origins manifested impaired growth irrespective of growth conditions and enhanced cell size, and exhibited excessive and asynchronous replication initiation. The generation time of an *E. coli* strain with three replication origins decreased in a minimal medium supplemented with glucose as the sole carbon source. As well as cell growth, the introduction of additional replication origins promoted increased biomass production.

**Conclusions:**

Balanced cell growth and physiological stability of *E. coli* under rapid growth condition are affected by changes in the position and number of replication origins. Additionally, we show that, for the first time to our knowledge, the introduction of replication initiation sites to the chromosome promotes cell growth and increases protein production.

**Supplementary Information:**

The online version contains supplementary material available at 10.1186/s12934-022-01851-z.

## Background

DNA replication is initiated at a specific nucleotide sequence called the origin of replication. The accuracy and speed of genome replication are essential for efficient cell division. Chromosomal DNA replication plays a major role in transferring genetic material to the next generation. Most bacteria have a circular genome with one replication initiation site, which is called *oriC*. Archaea use conserved replication origins like bacteria [1], but previous studies have reported that chromosomal replication can be initiated at several loci on the chromosome [1–3]. The location of eukaryotic replication initiation depends on the structural properties of DNA and its topology, not on the consensus DNA sequence except for *Saccharomyces cerevisiae* and *Saccharomycotina* species [4–6]. Furthermore, genome sequencing-based determination of the replication origin showed the repeated G-rich motifs near the replication initiation site in metazoans [7]. However, the aspect of the replication origin is not only different between species, but also can changed during cell differentiation and development [6].

*Escherichia coli* has a circular chromosome with a single *oriC* and a terminus of replication (*ter*), which is located opposite to *oriC*. *ter* sites pause the replication fork to avoid over-replication in the leading strand by interacting with the terminator protein Tus, which binds to the termination sites [8–12]. The Tus*-ter* complex blocks the progression of the replication fork in a direction-dependent manner (permissive or non-permissive face) [13]. On the other hand, in the *dif* site, recombination site in the opposite direction of *oriC*, chromosome dimers are resolved to monomers by XerC and XerD [14, 15]. Expression patterns of the regulatory genes coincide with the order of genes along the replichores and the relative distance of these genes from *oriC* is highly conserved in 131 γ-proteobacterial genomes [16]. The expression of genes is influenced by their location relative to *oriC* on the *E. coli* chromosome. DNA replication can induce changes in the promoter activity and the copy number, which gradually decrease based on its position from *oriC* to *dif* [17]. Therefore, the unique location of *oriC* is critical for chromosomal DNA replication and bacterial physiology, because its position is highly associated with the expression levels of genes that act as global regulators and their targets.

The cell cycle of *E. coli* is generally divided into the following three periods: B period, the time between cell division and replication initiation; C period, the time required to complete DNA replication; and D period, the time required for cell division [18]. DNA replication in the cell cycle is a highly regulated process that ensures the correct and complete chromosome replication before the division of the daughter cells from the parent cells. The binding of the DnaA protein, which unwinds the double-stranded DNA, initiates DNA replication at *oriC* and facilitates the recruitment of the replisome complex that is composed of replication components such as helicase, primase, and DNA polymerase III [19].

In a nutrient-rich medium, the *E. coli* cells can have a multi-forked chromosome during the exponential growth phase because of concurrent rounds of replication. Multiple pairs of active replication forks synthesize DNA from *oriC* to *ter*. Thus, the number of replication origins in rapidly dividing *E. coli* is 2^n^. The *E. coli* cells can contain four or eight replication origins at one time point during rapid cell division [20]. However, a small proportion of the cells (< 5%) with ongoing DNA replication can contain atypical numbers of chromosomes (such as 3, 5, and 7) due to asynchronous replication initiation [21]. Bacterial replication pattern is dependent on cell growth conditions [22, 23]. The growth-dependent accumulation of DnaA is reported to be the primary trigger for replication initiation in *E. coli*. However, increased DnaA concentration does not affect the timing of replication initiation [20, 24]. Therefore, the balance between cell growth and DNA synthesis is maintained by the proportional increase in the copy number of chromosomes based on the growth rate.

Several studies have analyzed the roles of replication origin in chromosomal replication by inserting an additional origin or deleting the original one. The integration of additional replication origin (*oriZ*), which consists of a 5.1-kb region containing the *oriC* sequence with five genes, *asnA, asnC, gidA*, *gidB*, and *mioC*, into the upstream region of *lacZ* resulted in similar growth compared to the parental strain *E. coli* AB1157 [25]. Also, the DNA replication of *E. coli* derivatives with an ectopic replication origin, which exhibited altered DNA synthesis, was correlated with transcription [26]. Another *E. coli* strain, which contains an ectopic origin *oriZ*, showed a marked variation in colony size and an increased doubling time, which is two times higher than that of the wild type [27, 28]. Asymmetric replication partially contributes to the increased doubling time as one replication fork progresses to replicate approximately 3.3 Mb of DNA, whereas the other fork progresses to replicate approximately 1.3 Mb of DNA [28]. In addition to bacteria, alterations in the number of replication origins influence the growth rate of an archaeon. The growth rate of *Haloferax volcanii*, which contains four replication origins, can be accelerated by removing the replication initiation sites [29].

In this study, *E. coli* strains with multiple or ectopic replication origins were constructed and their relative viability, specific growth rate, and cell volume were measured. Marker frequency and flow cytometry analyses were performed to assess the cell cycle parameters, such as the C and D periods. Replication profile analyses were performed to investigate the read counts across the whole genome and to verify the marker frequency analysis results. Moreover, an optimal culture condition for the recombinant *E. coli* strain was established to evaluate the reproducibility and repeatability of the improved growth rate of *E. coli* derivatives. Finally, the total protein amount in the *E. coli* derivatives relative to that in the wild-type MG1655 strain was measured to determine biomass production.

## Results

### **Construction of*E. coli*strains containing multiple or ectopic replication origins**

**Fig. 1:**
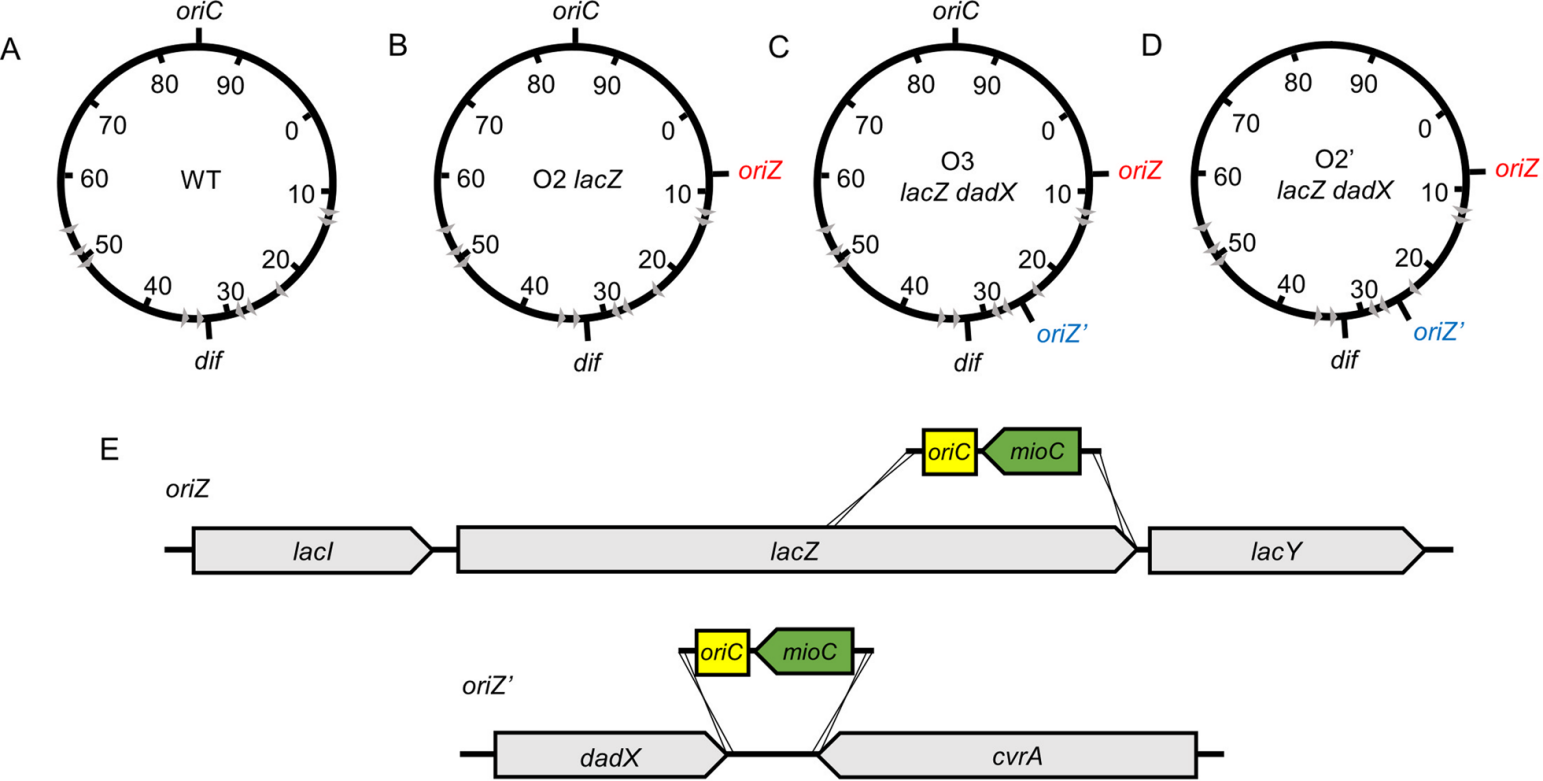
*Escherichia coli* derivatives with multiple or ectopic replication origins. The circles represent the chromosome of the *E. coli* MG1655 (4.6 Mb) and its derivatives. The gray triangle indicates the replication termination sites on the chromosome. **A** Wild-type MG1655. **B** O2 *lacZ* with two replication origins. **C** O3 *lacZ dadX* with three replication origins. **D** O2’ *lacZ dadX* with two ectopic replication origins. **E** Schematic presentation of the *oriZ and oriZ’* cassette integration

The effects of the number and position of replication origins on the physiology of *E. coli* MG1655 were examined. A previous study showed that the genomic region containing a partial sequence of *gidA*, *oriC*, and *mioC* can act as a replication origin [25]. Thus, we additionally eliminated the partial sequence of *gidA* when designing the recombination cassette, because the partial sequence may not operate as an intact protein or produce truncated protein. The native replication origin region, which included both *oriC* and *mioC*, was introduced into *lacZ* (365,214–363,275 bp) or the convergent intergenic region between *dadX* and *cvrA* (1,239,951–1,250,334 bp) using the λ Red and FLP/FRT recombination systems. This modification does not directly affect bacterial cell physiology [30]. The O2 *lacZ* and O3 *lacZ dadX* strains containing two or three native replication origins, respectively, were constructed (strains with multiple/additional replication origin(s), Fig. 1B, C). To examine the activity of the newly introduced ectopic replication origins and their positional effects on the timing of replication initiation, O3 *lacZ dadX* was used as a parent strain to generate strain with ectopic replication origins. The original 801-bp replication origin region, which included both *oriC* and *mioC*, of strains with multiple replication origins was replaced with the kanamycin resistance gene (*kan*). The genotypes of *E. coli* derivatives are listed in Additional file 1: Table S1. The strain with multiple/ectopic replication origins (O2’ *lacZ dadX*) contained two off-site replication origins (*oriZ* and *oriZ’*) at a distance of approximately 1 Mb and 2 Mb from the location of *oriC* (Fig. 1D). Thus, the strains with two or three replication origins and two ectopic replication origins without *oriC* were constructed in this study.

### **Cell viability and growth phenotypes of*E. coli*with aberrant replication origins**

The effect of replication origin (*oriC*-*mioC*) disruption on bacterial proliferation was examined using a bacterial cell viability assay. We used flow cytometry to measure the viability of each strain using a SYTO9 and propidium iodide fluorescent staining-based method [31]. The excitation/emission peaks are generated near 480/500 nm for SYTO9 and 490/635 nm for propidium iodide (Thermo Fisher Scientific). Generally, SYTO9 can label all bacteria regardless of their membrane integrity, but propidium iodide cannot penetrate intact bacterial membrane. Thus, by comparing the fluorescence in each cell, we could discriminate the viable and dead cells. Additionally, flow cytometry analysis was used since it can analyze a much larger number of cells at multiple wavelengths than fluorescence microscopy. Flow cytometry analysis was performed to count the number of live or dead cells in a population of 2 × 10^4^ cells. Cell viability, which was calculated as the ratio of live cells to dead cells, of the derivatives was compared with that of the wild-type MG1655 strain. The percent viabilities of O2 *lacZ* and O3 *lacZ dadX* were 97.1% and 96.8%, respectively (Additional file 1: Fig. S1A). Similarly, the percent viability of O2’ *lacZ dadX* was 97.0% (Additional file 1: Fig. S1B). Thus, the strains with multiple replication origins or ectopic replication origins and the wild type exhibited similar viability. This indicated that the proliferation rates of all *E. coli* derivatives in lysogeny broth (LB) were proportional to the fraction of live cells.

Next, the growth rates of wild-type MG1655 and its derivatives were determined in LB or M9 minimal medium supplemented with 0.4% glucose as the sole carbon source (Table 1). The growth rates of the strains with multiple replication origins were similar to that of the wild-type strain in LB. In contrast, the growth rate of the strain with ectopic replication origins was markedly lower than that of the wild-type strain in LB. The doubling time of O2’ *lacZ dadX* was 39.35 ± 0.93 min, which was 60.7% longer than those of wild-type cells (24.49 ± 0.38 min). However, the growth rates of O3 *lacZ dadX* and O2 *lacZ* were 7.4% and 4.4% faster than that of MG1655 (60.79 ± 0.10 min), respectively, in M9 medium with glucose. These results show that the growth rate proportionally increases as the number of replication origin increases when the original one is intact. However, the growth rate of O2’ *lacZ dadX* decreased by 11.8%, when compared with that of MG1655. These findings indicate that the introduction of additional replication origins can exert beneficial effects on bacterial growth under nutrient-limiting conditions. Also, the growth rate did not correlate with the number of replication origins and the physiological changes associated with cell growth are influenced by the position and not the number of replication origins.

**Table 1:** Differences in cell growth of *Escherichia coli* under nutrient-rich or -limiting conditions

Strain	Lysogeny broth			M9 medium with 0.4% glucose		
	Growth rate (h^− 1^)	Generation time (min)	Difference^a^ (relative ratio)	Growth rate (h^− 1^)	Generation time (min)	Difference^a^ (relative ratio)
MG1655	1.70 ± 0.03	24.49 ± 0.38	1.00	0.68 ± 0.01	60.79 ± 0.10	1.00
O2 *lacZ*	1.65 ± 0.02	25.26 ± 0.23	1.03	0.71 ± 0.01	58.74 ± 0.16	0.97
O3 *lacZ dadX*	1.64 ± 0.02	25.36 ± 0.28	1.04	0.73 ± 0.01	57.01 ± 0.12	0.93
O2’ *lacZ dadX*	1.06 ± 0.03	39.35 ± 0.93	1.61	0.60 ± 0.01	69.08 ± 0.87	1.13

Data are presented as mean ± standard error

^a^The growth rates of *E. coli* strains were normalized to that of wild-type MG1655

The growth rates of the wild type and O3 *lacZ dadX* were measured under different growth conditions to determine the reproducibility of enhanced cell proliferation (Additional file 1: Table S3). LB, LB with glucose, YT, and TB, which are nutrient-rich media, were used to promote rapid growth. A minimal medium with glucose or glycerol was used for slow growth. The growth rates of O3 *lacZ dadX* were similar in different nutrient-rich media. Additionally, the growth rate of *E. coli* strains in 200 µL M9 medium with glucose in a 96-well plate was measured every 20 min using a microplate reader. Compared with those in the MG1655 cells, the growth rate increased by 15.1% and the doubling time decreased by 13.1% in O3 *lacZ dadX* cells (Additional file 1: Table S4). To confirm the growth rate of each strain in a different setting, the cells were cultured in 50 mL medium in a 250 mL Erlenmeyer flask. Again, the growth rate increased by 9.7% and the generation time decreased by 8.9% in O3 *lacZ dadX* as compared to MG1655 (Additional file 1: Table S3). In summary, the growth rate of the strain with three replication origins was similar to that of the wild type under rapid growth conditions. However, its growth rate was faster than that of the wild type in M9 medium with glucose. Additionally, the culture volume affected the aeration during cell culture and resulted in different growth rate.

### **Growth-dependent morphological changes in*E. coli*with aberrant replication origins**

**Fig. 2:**
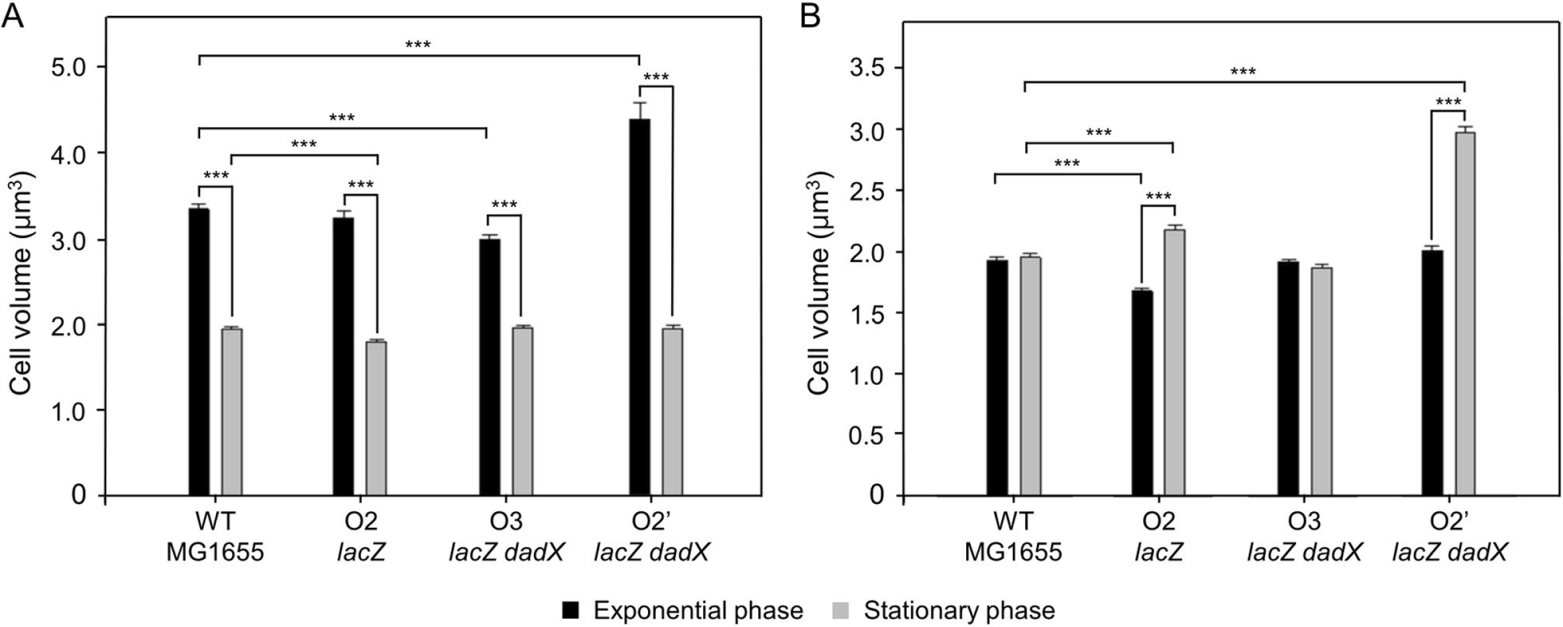
Cell size variation of *Escherichia coli* strains in different growth phases. Black or gray bars with standard errors indicated the cell volumes of the *E. coli* strains at exponential or stationary phase, respectively, in **A** LB or **B** M9 medium supplemented with 0.4% glucose (n = 200 in each experiment). ***P < 0.0001

Cell size (length and diameter) was measured, and cell volume was calculated at the exponential or stationary phase under different growth conditions to investigate the physiological response of *E. coli* derivatives to the changes in the position and number of replication origins. The cells in the exponential phase were inoculated into fresh media and the morphology of cells was examined at the exponential phase (Additional file 1: Fig. S2). The changes in the cell volume at different growth phases of *E. coli* derivatives were examined using Gram staining. The volume of the wild-type cells decreased from the exponential phase to the stationary phase in LB (Additional file 1: Table S5). The cell volume at the exponential phase was 41.7% higher than that at the stationary phase (Fig. 2A). Similarly, the volume of strains with multiple replication origins decreased from the exponential phase to the stationary phase. The volume of O3 *lacZ dadX* strain at the log phase decreased by 10.7% when compared with that of the wild-type strain. Additionally, the volume of the O2 *lacZ* strain at the stationary phase decreased by 7.9% when compared with that of the wild-type strain. In contrast, strain with ectopic replication origins exhibited significantly decreased proliferation irrespective of growth conditions, as well as heterogeneous and increased cell volume, when compared with the wild-type strain. The volume of O2’ *lacZ dadX* cells was 31.3% higher than that of MG1655. Meanwhile, the length of O2’ *lacZ dadX* was 35.0% higher than that of MG1655 (Additional file 1: Table S5).

In M9 medium with glucose, the volume of wild-type and O3 *lacZ dadX* strains did not change much from the exponential phase to the stationary phase (Fig. 2B). However, the O2 *lacZ* and O2’ *lacZ dadX* strains exhibited elongated morphology under the nutrient-deficient condition (Additional file 1: Fig. S2). At the stationary phase, the cell volume of O2’ *lacZ dadX* increased by 52.4%, when compared with that of the wild-type strain (Fig. 2B). These results indicate that the growth conditions markedly influenced the cell size.

### **Cell cycle analysis of*E. coli*with aberrant replication origins in LB **

**Fig. 3:**
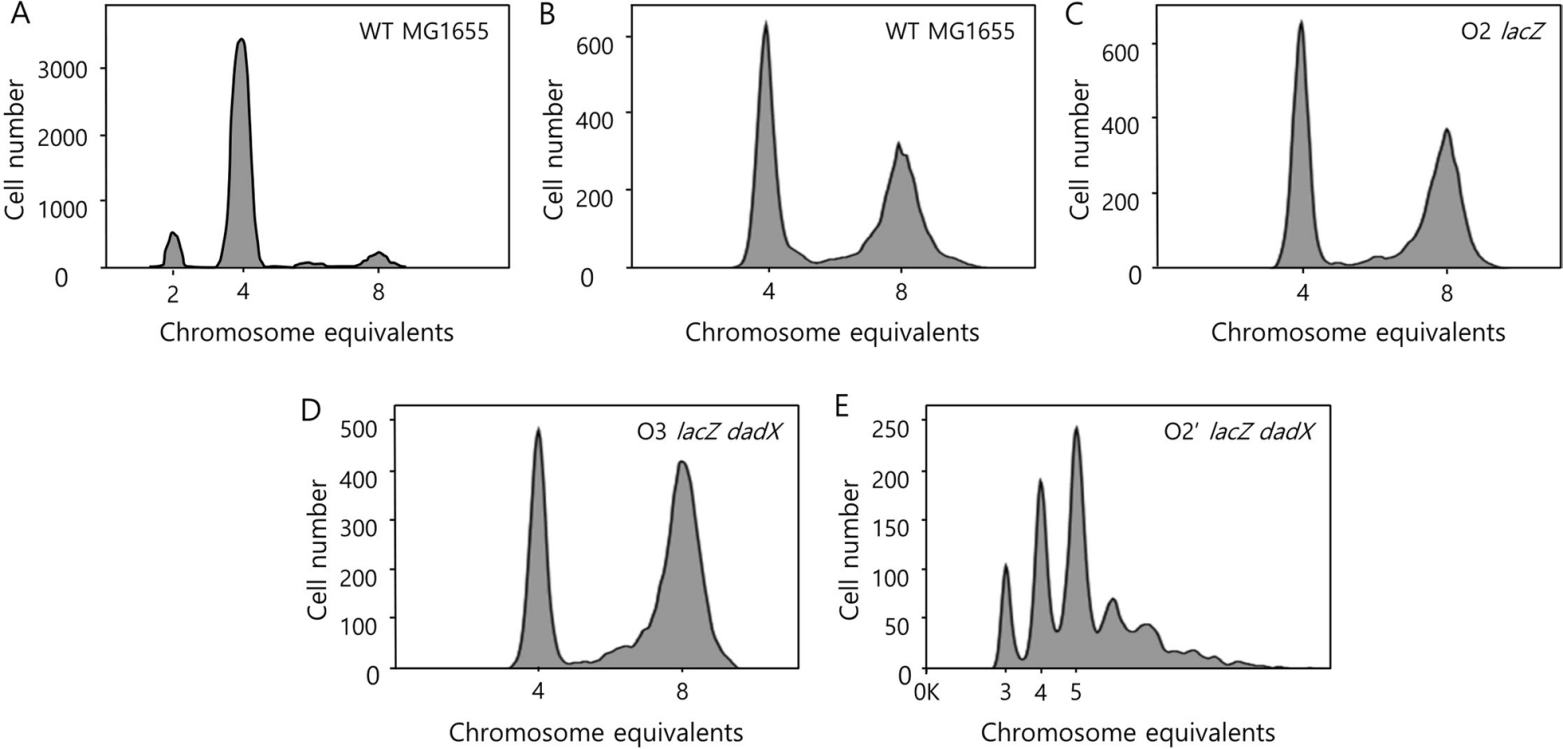
DNA replication run-out histograms of *Escherichia coli* derivatives in LB. Chromosome contents of each *E. coli* strain at the stationary phase (**A**) and exponential phase in LB (**B**–**E**) were determined using flow cytometry analysis. Two major peaks of Hoechst fluorescence indicated that *E. coli* cells contained four or eight chromosomes (**B**–**D**). Four to five major peaks with indistinguishable minor peaks of Hoechst fluorescence indicated that *E. coli* cells contained several copies of the chromosomes (**E**). **A** Wild-type cells in the stationary phase. **B** Wild-type cells in the exponential phase. **C** O2 *lacZ* cells in the exponential phase. **D** O3 *lacZ dadX* cells in the exponential phase. **E** O2’ *lacZ dadX* cells in the exponential phase

Flow cytometry analysis was performed to demonstrate the effects of additional and ectopic replication origins. *E. coli* derivatives were sampled at the early exponential phase and treated with rifampicin and cephalexin to block new replication initiation and cell division. Combination of two antibiotics leads to the completion of in-progress replication. As a result, integral numbers of completely replicated chromosomes can exist in a single cell. Thus, measurement of the number of chromosomes in this sample stands for the number of actively replicating replisomes at a time. The left-hand peak of the DNA replication run-out histogram corresponds to the fraction of the cells with four chromosomes in which replication was not initiated (Fig. 3B). In contrast, the peak on the right corresponds to the fraction of cells with eight chromosomes, in which replication was initiated when *E. coli* derivatives were cultured in LB [20]. The DNA replication run-out histograms revealed that the culture of strains with multiple replication origins contained an increased proportion of replicating chromosome (Fig. 3). As a result, the fraction of replicating chromosome in the culture of O2 *lacZ* was similar to that in the culture of MG1655. However, the fraction of cells with eight chromosomes in the culture of O3 *lacZ dadX* was 27.5% higher than that in the culture of MG1655 (Fig. 3B–D). Compared with those in MG1655, the number of origins per cell increased by 9.3% and the replication initiation period decreased by 32.0% in the O3 *lacZ dadX* cells (Additional file 1: Table S6). However, the duration of the C and D periods in the O3 *lacZ dadX* strain increased by 5.0% when compared with that in MG1655. In contrast, several peaks were observed in O2’ *lacZ dadX*, which exhibited asynchronous and excessive replication initiation when compared with MG1655 (Fig. 3E). Hence, cell cycle parameters, such as C and D periods, the number of origins, and initiation age of O2’ *lacZ dadX,* were excluded. In conclusion, asymmetric replication in the strain with ectopic replication origins leads to a larger deviation in the pattern of DNA replication as compared to the wild type.

### **Replication profile analysis of*E. coli*with aberrant replication origins**

The replication profiles of the wild type, O2 *lacZ*, and O3 *lacZ dadX* were comparatively analyzed to examine the effects of *oriZ* and *oriZ’* addition in LB (Additional file 1: Fig. S3). Genomic DNA samples were isolated from *E. coli* cells treated with sodium azide during logarithmic growth. The reference genomic DNA was prepared after treatment with rifampicin and cephalexin. The sequence reads were aligned to the reference genome and the number of sequence reads with a size of less than 1000 bp was calculated to determine the average coverage. The replication profile analysis of MG1655 confirmed bidirectional replication from *oriC* to *terC*. Average enrichment for MG1655 was the highest in the replication origin region at *oriC* and the lowest in the terminus region at *terC* (Additional file 1: Fig. S3A). In O2 *lacZ*, the enrichment pattern shows the peak at the original *oriC* and *oriZ* regions (Additional file 1: Fig. S3B). Also, the genomic region of *oriZ*’ in O3 *lacZ dadX* slightly increased as compared to O2 *lacZ* (Additional file 1: Fig. S3C). Generally, strains with additional replication origins show lower enrichment near *oriC* region compared to the wild type.

In M9 minimal medium with glucose, the replication profile of MG1655 shows sparse distribution of enrichment across the genome as compared to that in LB (Additional file 1: Fig. S3D). Also, the enrichment ratio of *ori* and *ter* was decreased. Generally, the growth rate of the cells is correlated with the replication rate (*ori*/*ter*) of the cell [32]. Thus, the slow growth of wild-type *E. coli* in M9 medium with glucose seems associated with the reduced replication rate. In the case of O3 *lacZ dadX*, its replication profile appeared more dispersed as compared to MG1655 (Additional file 1: Fig. S3E). However, we could observe replication initiation at the *oriZ* and *oriZ’* regions in the chromosome. Although enrichment in *oriC* is similar between MG1655 and O3 *lacZ dadX*, replication initiation from the additional replication origins may have contributed to accelerated growth in M9 minimal medium with glucose.

**Fig. 4:**
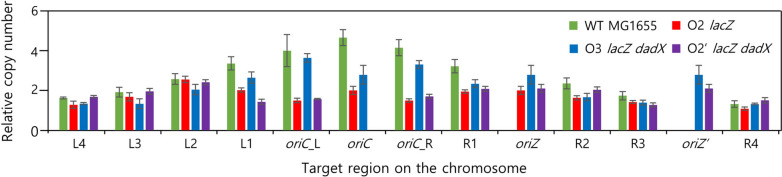
Chromosomal copy numbers of each locus for exponential cell populations in LB. The quantitative real-time PCR analysis was performed to measure the location-dependent copy number variation in the target regions of strains with multiple or ectopic replication origins. Data are presented as mean ± standard error. Both *oriZ* and *oriZ’* target regions represent the positions located near the newly introduced ectopic sites, *lacZ* and intergenic region between *dadX* and *cvrA*. Green, red, and blue, and purple bars indicate the wild type, O2 *lacZ*, and O3 *lacZ dadX*, and O2’ *lacZ dadX*, respectively. Copy numbers were calculated from triplicated experiments

Next, we performed marker frequency analysis to measure the amount of time spent during the C and D periods in *E. coli* derivatives. The C period was calculated using the quantitative real-time polymerase chain reaction (qRT-PCR) results of the *oriC*/*terC* ratio in early exponential phase cells. The D period, which was measured as the number of origins per cell, was examined using flow cytometry analysis. The equation used to calculate the C and D periods is described in the Methods section. Twelve specific primer sets were designed to measure the variation in copy numbers of regions at every 500 kb position from *oriC* to *terC* along the right and left replichores bi-directionally. The copy number of each site in the wild-type cells gradually decreased from *oriC* to *terC* (Fig. 4). This result was consistent with that of a previous study, which reported that the copy numbers of the left replichore were higher than those of the right replichore [33]. The *oriC*/*terC* ratio indicated that the copy numbers in the wild-type, O2 *lacZ*, and O3 *lacZ dadX* strains were 4.71 ± 0.40, 2.03 ± 0.21, and 3.10 ± 0.55, respectively, in LB. The *oriC*/*terC* ratio in the O2 *lacZ* strain decreased by 57.0% when compared with that in the wild-type strain. The copy number in the O3 *lacZ dadX* strain recovered by 22.8% when compared with that in the O2 *lacZ* strain. In contrast to strains with multiple replication origins, the copy number in O2’ *lacZ dadX* was 2.13 ± 0.21. For O2 *lacZ* and O2’ *lacZ dadX* cells, copy numbers of the L2 region in the left replichore were higher than other regions (Fig. 4). The marker frequency analysis results revealed that the activity of the ectopic replication origins containing *oriC* and *mioC* was sufficient to initiate DNA replication and that the copy number deviation of the specific region appeared in the left replichore.

### **Biomass production of*E. coli*O3*lacZ dadX*in a minimal medium**

**Fig. 5:**
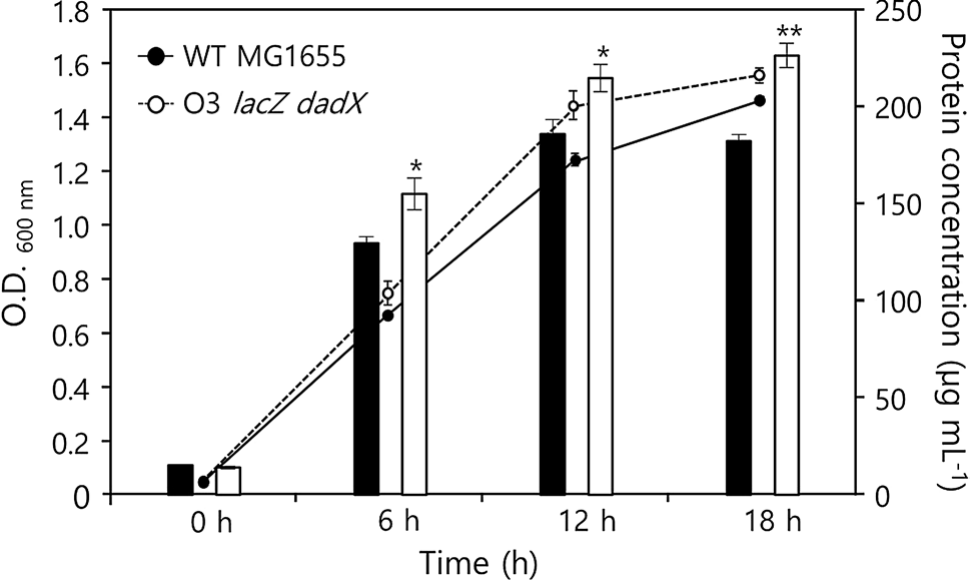
Growth-dependent increase of the biomass of *E. coli* O3 *lacZ dadX*. Total proteins were extracted and each optical density for Bradford assay was measured by every 6 h for each *E. coli* strain. Black and white bars with standard errors indicated as the total protein concentration for the wild type and O3 *lacZ dadX*, respectively. A single or double asterisk indicates *p*-value either under 0.05 or 0.01. Bradford assay and growth curve measurement were performed triplicated

We then measured the biomass productivity using the wild type and O3 *lacZ dadX* to demonstrate the consequences of growth acceleration. The wild-type and O3 *lacZ dadX* strains exponentially growing in M9 medium with glucose as the sole carbon source were inoculated into a fresh medium. Cell growth and total protein concentration, which were used as parameters to determine biomass, were measured every 6 h for 18 h. At each time point, cell growth and total protein concentration varied. The biomass concentrations in O3 *lacZ dadX* at 6, 12, and 18 h were 155.42 ± 8.16, 215.47 ± 7.62, and 227.08 ± 6.28 µg mL^− 1^, respectively, whereas those in the wild type were 130.02 ± 3.22, 186.46 ± 7.62, and 182.93 ± 3.28 µg mL^− 1^, respectively (Fig. 5). The maximum biomass concentration in O3 *lacZ dadX* cells was achieved at 18 h, which was 24.1% higher than that in the wild-type strain. This indicated that biomass concentration was additionally increased as the growth of the cell accelerated.

## Discussion

This study examined the physiological roles of the *oriC*-*terC* axis by altering the number and locations of the replication origin. *oriC* is a major factor that mediates DNA synthesis and bacterial cell cycle. Hence, we hypothesized that alterations in the number or position of *oriC* can affect the usage of replication origins and consequently the efficiency of DNA replication. These alterations in the number or position of *oriC* can promote rapid growth and enable the utilization of multiple replication initiation systems (a mechanism observed in eukaryotic DNA) in *E. coli*. The importance of the location and number of replication origins was demonstrated using three *E. coli* strains with multiple or ectopic replication origins through physiological and cell cycle analyses. The *E. coli* strains constructed in this study exhibited distinct characteristics. Although the viability of *E. coli* and its derivatives was not affected, the doubling time of the O2’ *lacZ dadX* strains markedly increased irrespective of the growth conditions. This may be attributed to the asymmetric pattern in both replichores (one replication fork must replicate three quarters, while other forks replicating a quarter of the chromosome). In contrast to that in the strain with ectopic replication origins, the average growth rate of the strain with three replication origins (O3 *lacZ dadX*) increased, on average, by 8.3% in M9 minimal medium supplemented with glucose as the sole carbon source. Moreover, the biomass concentration was directly proportional to cell growth under nutrient-limiting conditions. On the other hand, O3 *lacZ dadX* showed growth characteristics similar to the wild type in several nutrient-rich media.

To understand the role of replication initiation in cell cycle, flow cytometry and marker frequency analyses were performed to determine the time required to complete replication (C period) or cell division (D period) in LB medium. Previous studies have reported that earlier replication initiation increases the duration of the C period. Conversely, the delay in replication initiation decreases the duration of the C period [20]. The copy numbers of the left replichore were reported to be higher than those of the right replichore. Additionally, left replichore replication precedes the right replichore replication by up to 7 min [33]. Flow cytometry analysis revealed that the deviation in DNA replication and cell division in O2’ *lacZ dadX* was due to asynchronous DNA replication based on the chromosome equivalent between 4 and 8. The culture of strains with multiple replication origins exhibited an increased fraction of replicating chromosome.

Asymmetric replication in strain with ectopic replication origins markedly affected fork progression as the left replichore of the two replication forks from the ectopic replication origin should progress approximately twice as far as the right fork to complete DNA replication. These results implied that the initiation of DNA replication in strain with double ectopic replication origins is compromised due to the relocation of the replication origin to an ectopic site. However, the activity of ectopic replication origins, which contain *oriC* and *mioC*, was sufficient to initiate DNA replication. In contrast, the specific growth rates of strains with multiple replication origins were similar. The average C + D period and the number of *oriC* per cell in the cells with multiple replication origins, which were proportional to the fraction of replicated cells, were higher than those in the wild-type strain. Marker frequency analysis revealed that the O2 *lacZ* and O3 *lacZ dadX* strains had decreased copy number at 12 loci compared to the wild type. Therefore, positional effects are considered to induce over-initiated replication, as well as to delay replication initiation.

Direction-dependent permissiveness of the Tus-*ter* complex can affect the progression of the replication fork [9]. Thus, integration of the replication origin to other sites of the chromosome can alter the direction of replication on termination sites. For example, if the replication origin is inserted into the locus between *terC* and *dif* or *terA* and *dif*, direction of replication will be reversed to the permissive direction. By characterizing the cellular aspects (e.g., physiological traits or replication fork progression) in these strains and their derivatives without Tus, the implications of unidirectionality of the Tus-*ter* barrier could be studied.

The cell volume in the log phase was larger than that in the stationary phase. The cell volumes at the stationary phase in LB were constant for *E. coli* strains with multiple replication origins, except for the O2 *lacZ* strain. The reduction in cell volume from the exponential to stationary phase was correlated with the growth rate, which declined over time in LB. However, the cell volume and growth rate were not correlated in the minimal medium, which can be attributed to the limited growth rate. The introduction of ectopic replication origins and the inactivation of the native replication origin promoted heterologous changes and increased cell volume in the cultures irrespective of growth conditions. In particular, the volume of strain with ectopic replication origins was significantly influenced by changes in genomic orientation. Consequently, the results of DNA replication run-out experiments revealed that cell size variation was dependent on the timing of replication initiation.

Although a previous study showed increased cell growth after the removal of the replication origins in archaea [29], deletion of the original replication origin in *E. coli* and *Bacillus subtilis* had adverse effect on growth [34]. However, insertion of an additional replication origin to a specific site resulted in increased biomass production in M9 minimal medium with glucose, but not in nutrient-rich medium. This may be affected by the lack of proportional increase in the production of DnaA because the activity of the replication origin is strictly regulated and limited to once per cell cycle by regulatory inactivation of DnaA (RIDA) [35]. Thus, stoichiometric analysis may be considered to define the ratio of DnaA^ATP^ and DnaA^ADP^ in the cells with multiple or ectopic replication origin.

*E. coli* cells are useful cell factories for metabolite and protein production. Metabolic engineering and introduction of a gene via the plasmid system have been used to produce these molecules [36, 37]. Generally, *E. coli* K strains are used to clone nucleic acids and produce metabolites, while B strains usually are used to over-express recombinant proteins [38]. B strains produce more protein because of their ability to synthesize amino acids and reduced protein degradation compared to K strains. However, *E. coli* K is more resistant to various environmental stresses. Thus, by increasing the protein production in K, stress tolerant protein cell factory can be constructed. Moreover, the growth of cells is crucial for molecular synthesis of proteins or metabolites, because the ribosomal fraction increases as the growth rate increases, and translation process is a limiting factor for protein synthesis [39, 40]. Nevertheless, nutrients can be devoted to cell growth rather than production of target molecules. Thus, to demonstrate the production of a specific target molecule, introduction of genes related to carbon-based metabolite synthesis (e.g., 3-hydroxypropionic acid) or protein should be considered. In addition, the relocated replication origin can induce head-on replication-transcription encounters that occur, because DNA polymerase and RNA polymerases share same DNA as the template. Although the overall co-directionality between replication and transcription in *E. coli* is known to be 54%, co-directionality of highly transcribed genes coding ribosomal proteins such as *rrn* C, A, B and D is 93% [41, 42]. The interference where occur in highly transcribed areas on the *E. coli* chromosome during replication and transcription can be problematic and interrupt the progression of replication forks [34, 43]. Replication-transcription conflict is known to increase the mutation rate and critical factors for balanced cell growth and physiological stability [28, 44]. Thus, genetic stability should be analyzed by calculating the mutation rate or tracking adaptive mutations occurring in a specific region through experimental evolution of O3 *lacZ dadX*. Further, to identify the mechanism of this growth acceleration, multi-omics analysis including transcriptome and proteome analysis could be considered.

## Conclusions

This study demonstrated that the insertion of a new replication initiation site in *E. coli* can significantly influence bacterial growth, replication rate, cell cycle, and morphological features. Additionally, the insertion of an additional replication initiation site can accelerate the growth rate of *E. coli* in M9 minimal medium supplemented with glucose. This study suggestes a novel mechanism to improve the growth rate and protein production in *E. coli*.

## Materials and methods

### Strains and growth conditions

In this study, *E. coli* K-12 MG1655 [F^−^ lambda^−^ *ilvG rfb*-50 *rph*-1 *glpK*(G184T)] was used. The *E. coli* derivatives were cultured in LB or minimal M9 medium supplemented with 0.4% glucose as the carbon source at 37 °C with a shaking. The composition of M9 solution was as follows: 33.9 g Na_2_HPO_4_, 15 g KH_2_PO_4_, 5 g NH_4_Cl, and 2.5 g NaCl (prepared in 1 L distilled water). The following components were sterilized and added to the M9 minimal medium: 200 mL of M9 solution, 2 mL of 1 M MgSO_4_ solution, 0.1 mL of 1 M CaCl_2_ solution, and 20 mL of glucose (20%) (for 1 L medium). The composition of the LB medium was as follows: 10 g tryptone, 5 g yeast extract, and 5 g NaCl (prepared in 1 L distilled water) and supplemented with antibiotics (100 µg/mL ampicillin or 50 µg/mL kanamycin). The antibiotics were used to select the *E. coli* MG1655 and its derivatives. Nutrient-rich media (LB, LB supplemented with 0.4% glucose, YT, and terrific broth (TB) medium) or minimal media (M9 medium supplemented with 0.4% glucose or 0.2% glycerol) were used to test the repeatability and reproducibility of the cell growth rate.

### Selection of ectopic sites

The replication origin was introduced in the coding region of *lacZ* (*oriZ*) and the convergent intergenic region between *dadX* and *cvrA* (*oriZ*’) in the *E. coli* MG1655 genome, which did not result in adverse effects on growth or physiology. Genomic properties, including the orientation of genes, location of promoters, and binding sites of transcription factors were elucidated using the EcoGene (http://www.ecogene.org/), EcoCyc (https://ecocyc.org/), and RegulonDB (http://regulondb.ccg.unam.mx/) [45–48].

### **Genetic manipulation of*E. coli***

*oriC-mioC* and *oriC* deletion cassettes were used to integrate a native replication origin into the ectopic loci and delete the original replication origin. *oriC* and *mioC* were PCR-amplified from the genomic DNA of *E. coli* MG1655. The kanamycin resistance gene with two flippase (FLP) recognition target (FRT) sequences was amplified from pKD4 [30]. The *oriC-mioC* cassette comprised the replication origin of chromosome (*oriC*), the *mioC* gene, a selectable kanamycin resistance gene, two FRT sites, and two homology extension sequences (35 bp). The *oriC* deletion cassette comprised two homology extension sequences (50 bp) and a kanamycin resistance gene [26]. The insertion of *oriC*-*mioC* or *oriC* deletion cassette into the target regions, such as *lacZ*, intergenic region, and native replication origin was performed using λ Red recombination with the temperature-sensitive plasmid pKD46 expressing the λ Red proteins (*γ*, *β*, and *exo)* [30]. The recombinant *E. coli* cells harboring pKD46 were cultured at 30 °C. The expression of λ Red proteins was induced using 10 mM L-arabinose. The target regions were directly disrupted by transforming the *oriC-mioC* or *oriC* deletion cassettes. The recombinant *E. coli* cells were selected on L agar plates containing antibiotics and the genotype was confirmed using direct colony PCR. After the curing step of pKD46, kanamycin resistance genes of *E. coli* derivatives with multiple replication origins were eliminated using FLP/FRT recombination through the transformation of the FLP helper plasmid pCP20. The pCP20 plasmid expresses FLP recombinase that acts on the repeated FRT sites flanking the resistance gene. Next, pCP20 was eliminated by culturing the cells at 42 °C.

### Growth rate analysisrowth rate analysis

For Erlenmeyer flask culture, each strain was inoculated into 3 mL of LB or M9 minimal medium supplemented with 0.4% glucose and cultured overnight at 37 °C. The optical density at 600 nm (OD_600nm_) was measured using an OPIZEN POP spectrophotometer (Mecasys, Korea). The OD_600nm_ value of the stationary phase cultures of *E. coli* was diluted to less than 0.05 in 50 mL of fresh medium. The diluted samples were cultured to an OD_600nm_ value of less than 0.5. The amount of inoculum for determining the initial OD_600nm_ at time zero was calculated based on the OD_600nm_ value of 0.05 in 50 mL fresh growth medium. The exponential phase cultures were then diluted in fresh growth medium and cultured to an OD_600nm_ value of 0.3 for growth rate analysis. Therefore, *E. coli* cells in the exponential phase were used to determine the growth rate. Growth curve analysis of *E. coli* MG1655 and its derivatives was performed by measuring the OD_600nm_ of triplicate cultures. The OD_600nm_ values of exponential phase *E. coli* cultures were recorded at a range of 0.05–0.3 to calculate the specific growth rate [49]. The specific growth rate (µ), which was defined as the linear slope in a plot of ln (OD_600nm_) versus time (h), was calculated using the following equation:

$${\text{Specific}}\,{\text{growth}}\,{\text{rate}}\,\left( {{\mu }} \right) = \frac{{{\text{In}}\left( {N/N_{0} } \right)}}{t}$$

where *N* or *N*_0_ is the optical density of *E. coli* cells in a flask at the time in the exponential phase or at time zero, respectively, and *t* is the cultivation time [50]. Additionally, the specific growth rates of wild-type and O3 *lacZ dadX* strains from triplicate samples were verified using a plate reader (Epoch 2 microplate spectrophotometer; BioTek, USA). The following settings were employed for OD measurements: interval time, 20 min; shaking speed, 330 cpm; wavelength, 600 nm; temperature, 37 °C; temperature gradient, 1 °C; read speed, normal; read delay, 100 ms; number of measurements/data point, 8. The log phase *E. coli* culture was re-inoculated into 200 µL of fresh culture medium in 96-well plates. The OD_600nm_ was measured using a microplate reader to determine the specific growth rates using the same processes as described above.

### Cell morphology analysis

The stationary or exponential phase *E. coli* cultures were harvested and subjected to Gram staining [51]. The stained *E. coli* cells were observed using a Nikon Eclipse Ci-E microscope under a 100X immersion oil lens (NA 1.45). The images were captured using NIS-Elements software. Cell length (l) and width (*w*) were analyzed using the image analysis program ImageJ with the ObjectJ plugin (Object-Image version 2.21) [52]. The length and width of 200 individual cells were measured under each growth condition. For cell volume (V) measurements, the cells were assumed to have a cylindrical structure capped by two hemispherical ends as the dividing cells only exhibited an “8” morphology. Cell volume was calculated by measuring the length and width of dividing cells using the following equation [53]:

$${\text{Cell}}\,{\text{volume}}\,\left( {\text{V}} \right) = \frac{{\left[ {\pi \times w^{2} \times \left\{ {1 - \left( {\begin{array}{*{20}c} w \\ 3 \\ \end{array} } \right)} \right\}} \right]}}{4}$$

### Cell viability analysis

Cell viability was determined using the Live/Dead BacLight bacterial viability kit (Molecular Probes, L7012). The *E. coli* cells were cultivated in a 250 mL flask containing 50 mL of LB medium until the late log phase. The bacterial culture (25 mL) was centrifuged at 1,000 *g* for 15 min and the pellet was resuspended in 2 mL of 0.85% NaCl. Next, 1 mL of *E. coli* suspension was mixed with 20 mL of 0.85% NaCl (for live cells) or 70% isopropanol (for dead cells) in 50 mL Falcon tubes and incubated for 1 h. The samples were then centrifuged at 1000 g for 15 min. The pellet was resuspended and aliquoted into separate tubes containing 10 mL of 0.85% NaCl. The OD_600nm_ was measured to determine the number of *E. coli* cells. The *E. coli* suspension was mixed in different ratios of live cells to dead cells (100:0, 50:50, and 0:100) to a volume of 3 mL and the number of cells was adjusted to 1 × 10^7^ cells/mL (~ 0.01 OD_600nm_). The *E. coli* suspension (3 mL) containing live or dead cells was incubated with 6 µL of the SYTO9 (3.34 mM) and propidium iodide (20 mM) (1:1) mixture, which bind to DNA and RNA and emit fluorescent signal, at room temperature in the dark for more than 15 min. The number of live or dead cells was calculated using flow cytometric analysis. The SYTO9 and propidium iodide fluorescence intensities were measured at emission wavelengths of were measured at 538 and 620 nm, respectively, and an excitation wavelength of 485 nm using a flow cytometer (BD LSR II cytometer, BD Biosciences). The relative bacterial cell viability was then obtained using the following equation:

$${\text{Bacterial~cell~viability~}}\left( {\text{\% }} \right) = \left( {{\text{~}}\frac{{{\text{the~number~of~live~cells}}}}{{{\text{the~number~of~live~and~dead~cells}}}}~} \right) \times 100$$

### Flow cytometry analysis

The number of origins per cell was determined using the replication run-out method [54]. The fresh stationary phase cultures were diluted 200-fold to an OD_600nm_ of 0.01 and cultured to an OD_600nm_ of 0.4 in LB medium. For replication run-out, the exponential phase culture was incubated with rifampicin (150 µg/mL; Sigma-Aldrich, USA) and cephalexin (10 µg/mL; Sigma-Aldrich, USA) to prevent replication initiation and cell division for at least 3 h and chilled on ice for 5 min. All steps were performed at 4 °C. The cultures (2 mL) of the wild type or its derivatives were centrifuged at 300 g for 15 min. The pellet was washed twice with 1 mL Tris-EDTA (TE) buffer (10 mM Tris HCl (pH 7.4) and 10 mM EDTA), resuspended in 0.1 mL TE buffer, and fixed with 0.9 mL of 77% ethanol at − 20 °C overnight or longer. The fixed sample was washed twice with 1 mL of TM buffer (10 mM Tris HCl (pH 7.4) and 10 mM MgSO_4_). The OD_600nm_ of the cell suspensions was then determined. The final volume of the cell suspension (1 × 10^6^ cells) was adjusted to 0.5–1 mL in TM buffer. Next, the cells were incubated with Hoechst 33,342 (Molecular Probes, USA) solution in TM buffer (final concentration of 0.5 µg/mL) for at least 30 min in the dark. Flow cytometry was performed using a flow cytometer (BD LSR II cytometer; BD Biosciences, USA). A Coherent Sapphire 488 nm laser was used to generate forward and side scatter signals, while a Lightwave Xcite 355 nm (UV) laser was used to generate fluorescence signals. Additionally, a 505 LP dichroic mirror and a 440/40 band-pass filter were used to detect Hoechst 33,342 fluorescence [55]. Raw data (fcs3.0) were transformed and analyzed using FlowJo (version 10.2).

### Marker frequency analysis

The copy numbers of *oriC* and specific regions from *oriC* to *terC* were determined using qRT-PCR analysis. The delta delta CT (2^−ΔΔCT^) method was used for relative quantification [56, 57]. The *E. coli* cells for analyzing replicating genomic DNA samples were cultured to an OD_600nm_ of 0.4, treated with sodium azide (30 µg/mL; Fluka BioChemika, Switzerland), and incubated for 3 h at 37 °C. Genomic DNA was extracted using the Wizard® genomic DNA purification kit (Promega) and amplified using qRT-PCR system (ABI 7300; Applied Biosystems, USA) with premix (iTaq™ Universal SYBR® Green Supermix; Bio-Rad, USA). Twelve loci that included proximal regions for both *oriC* and *terC*, as well as specific priming regions per 500 kb from the origin (*oriC*) to terminus (*terC*) along the right and left replichores were used to profile the growth-dependent differences in the copy numbers in each priming region (see Additional file 1: Table S2). Cycle threshold (Ct) obtained from wild-type *E. coli* or its derivatives was analyzed using the 2^−ΔΔCt^ method. Marker frequency ratios were normalized to the *oriC*/*terC* ratio of each reference genomic DNA sample from *E. coli* cells treated with rifampicin (150 µg/mL; Sigma-Aldrich, USA) and cephalexin (10 µg/mL; Sigma-Aldrich, USA) [20].

### Replication profile analysis

The read counts across the genome of *E. coli* strains were determined using replication profile analysis. Genomic DNA from cultures of *E. coli* strains was extracted as described in the marker frequency analysis and subjected to next-generation sequencing. The genomic DNA was sequenced using Illumina HiSeq™ (50 or 100 bp single reads) or NovaSeq at Macrogen, Korea. The sequencing reads were imported into the CLC Genomics Workbench 9.0 (QIAGEN Bioinformatics, Germany) and assembled to the reference chromosome. The assembly was performed with the following parameters: 0.01 trim using quality scores; 15 discard reads below length; 2 mismatch cost; 3 insertion cost; 3 deletion cost; 0.5 length fraction; and 0.8 similarity fraction. Assembly data were mapped to the reference chromosome. The CSV files of the resulting mapping data were exported, and the average coverage per 1000 bp was calculated.

### Measurement of cell cycle parameters

The ratio of *oriC*/*terC* ratio was determined using the marker frequency analysis described above. The C period was calculated using the following equation [24]:

$${\text{C~period}} = \frac{{\ln \left( {\frac{{oriC}}{{terC}}} \right) \times \tau }}{{\ln \left( 2 \right)}}$$

where $\tau$ is the generation time of each *E. coli* strain in the exponential phase. Furthermore, the chromosome number at the exponential phase was determined using flow cytometry analysis as described above. The exponential phase of *E. coli* culture in LB medium was assumed to contain a mixture of 4 N cells that have not initiated DNA replication and 8 N cells that have completed DNA replication. Each fraction of the DNA contents was multiplied by chromosome equivalents (4 or 8) and added to determine the *oriC* per cell. The D period was calculated using the following formula:

$${\text{D~period}} = {\text{~}}\frac{{\ln \left( {\frac{{oriC}}{{{\text{cell}}}}} \right) \times \tau }}{{\ln \left( 2 \right)}} - {\text{C~period}}$$

The initial age (a_i_) was measured using the following equation [58]:

$${\text{a}}_{i} = - \frac{{(\ln \left( {1 - \frac{F}{2}} \right))}}{{\ln \left( 2 \right)}};{\text{~}}0 \le a_{i} \le 1$$

where *F* was the fraction of *E. coli* cells that have not initiated DNA replication.

## Supplementary Information

Below is the link to the electronic supplementary material.

**Additional file 1: Table S1.** *E. coli* strains used in this study. **Table S2.** Oligonucleotides used for genetic manipulation of *E. coli*. **Table S3.** Specific growth rates of *E. coli* MG1655 and O3 *lacZ dadX* under different growth conditions. **Table S4.** Reproducibility analysis of the enhanced growth rate of *E. coli* O3 *lacZ dadX*. **Table S5.** Growth condition-dependent changes in the cell size of *E. coli* derivatives cultured in LB or M9 medium supplemented with 0.4% glucose. **Table S6.** Summary of the flow cytometry analysis of *E. coli* cultured in LB medium. **Fig S1.** Relative cell viability of strains with multiple or ectopic origins in lysogeny broth. Bacterial cell viability was measured at the late log phase using flow cytometry. Cell viability of the strain was compared with that of wild-type MG1655. (A) Cell viability of O2 *lacZ* and O3 *lacZ dadX* with multiple replication origins. (B) Cell viability of O2’ *lacZ dadX* with ectopic replication origins. **Fig S2.** Growth-dependent changes in the cell morphology of *Escherichia coli* strains. The morphology of *E. coli* strains cultivated in LB or M9 medium with glucose was examined using Gram staining. Scale bar: 10 μm. **Fig S3.** Replication profiles of *Escherichia coli* strains. Replication profiles of (A) wild-type MG1655 in LB, (B) O2 *lacZ* in LB, (C) O3 *lacZ dadX* in LB, (D) MG1655 in M9 medium with glucose, (E) O3 *lacZ dadX* in M9 medium with glucose. Genomic DNA from the cultures of MG1655, O2 *lacZ*, or O3 *lacZ dadX* was extracted and subjected to next-generation sequencing as described in the Materials and Methods section. The average coverage for every 1000 bp in replicating sample DNA was normalized to reference DNA and plotted against each genomic position. The original replication origin (*oriC*), *oriZ*, *oriZ’*, and *ter* are shown in dotted lines.

## Data Availability

The sequences used in this study were deposited in Sequence Read Archive (SRA) under BioProject number PRJNA722362, which comprises 20 FASTQ files for Illumina HiSeq paired-end reads of MG1655 and O3 *lacZ dadX* in LB or M9 medium with glucose treated with sodium azide or rifampicin and cephalexin, as well as those of O2 *lacZ* in LB treated with sodium azide or rifampicin and cephalexin. The nucleotide sequences of plasmids, recombination cassettes, genomic loci near recombination site were deposited in SynBioHub under collection ID ME2022KK, which comprises nine GBK files for three plasmids, three recombination cassettes, and three genomic regions. All bacterial strains in this study are available with Material Transfer Agreement.
