# Supplementary material for: Escherichia coli cell factories with altered chromosomal replication scenarios exhibit accelerated growth and rapid biomass production
Source: Microb Cell Fact. 2022 Jun 21;21:125. doi: 10.1186/s12934-022-01851-z (PMC9210752; doi:10.1186/s12934-022-01851-z)
Supplement: Supplementary file 1 — Additional file 1: Table S1. E. coli strains used in this study. Table S2. Oligonucleotides used for genetic manipulation of E. coli. Table S3. Specific growth rates of E. coli MG1655 and O3 lacZ dadX under different growth conditions. Table S4. Reproducibility analysis of the enhanced growth rate of E. coli O3 lacZ dadX. Table S5. Growth condition-dependent changes in the cell size of E. coli derivatives cultured in LB or M9 medium supplemented with 0.4% glucose. Table S6. Summary of the flow cytometry analysis of E. coli cultured in LB medium. Fig S1. Relative cell viability of strains with multiple or ectopic origins in lysogeny broth. Bacterial cell viability was measured at the late log phase using flow cytometry. Cell viability of the strain was compared with that of wild-type MG1655. (A) Cell viability of O2 lacZ and O3 lacZ dadX with multiple replication origins. (B) Cell viability of O2’ lacZ dadX with ectopic replication origins. Fig S2. Growth-dependent changes in the cell morphology of Escherichia coli strains. The morphology of E. coli strains cultivated in LB or M9 medium with glucose was examined using Gram staining. Scale bar: 10 μm. Fig S3. Replication profiles of Escherichia coli strains. Replication profiles of (A) wild-type MG1655 in LB, (B) O2 lacZ in LB, (C) O3 lacZ dadX in LB, (D) MG1655 in M9 medium with glucose, (E) O3 lacZ dadX in M9 medium with glucose. Genomic DNA from the cultures of MG1655, O2 lacZ, or O3 lacZ dadX was extracted and subjected to next-generation sequencing as described in the Materials and Methods section. The average coverage for every 1000 bp in replicating sample DNA was normalized to reference DNA and plotted against each genomic position. The original replication origin (oriC), oriZ, oriZ’, and ter are shown in dotted lines. [file 12934_2022_1851_MOESM1_ESM.pdf]

# *Escherichia coli* cell factories with altered chromosomal replication scenarios exhibit accelerated growth and rapid biomass production

Hee Jin Yang[a], Kitae Kim[a], Soon-Kyeong Kwon[a,b], and Jihyun F. Kim[a*]

[a]Department of Systems Biology, Division of Life Sciences, and Institute for Life Science and Biotechnology, Yonsei University, 50 Yonsei-ro, Seodaemun-gu, Seoul 03722, Republic of Korea; [b]Division of Life Science, Gyeongsang National University, 501 Jinju-daero, Jinju-si, Gyeongsangnam-do 52828, Republic of Korea

Hee Jin Yang and Kitae Kim contributed equally to this work and share first authorship.

[*]Correspondence to: Jihyun F. Kim, Department of Systems Biology, Yonsei University, 50 Yonsei-ro, Seodaemun-gu, Seoul 03722, Korea. Tel: +82 2 2123 5561; Fax: +82 2 312 5657; E-mail: jfk1@yonsei.ac.kr.

# Supplementary Tables

**Table S1.** *E. coli* strains used in this study.

| Strain [a] | Genotype |
|---|---|
| MG1655 | Wild type (single *oriC*-*mioC*) |
| O2 *lacZ* | Δ*lacZ*::(*oriC*-*mioC*) |
| O3 *lacZ dadX* | Δ*lacZ*::(*oriC*-*mioC*) Δ(*dadX*-*cvrA*)::(*oriC*-*mioC*) |
| O2' *lacZ dadX* | Δ*lacZ*::(*oriC*-*mioC*) Δ(*dadX*-*cvrA*)::(*oriC*-*mioC*) Δ(*oriC*-*mioC*)::FRT-*kan*-FRT |

[a] The name of deleted regions is given for each *E. coli* strain. O2 and O3 represent strains containing the original *oriC* and multiple replications origins, which were introduced into each targeted region. O2' represent strain (derived from strains with multiple replication origins) with two ectopic replication origins, and contained a disrupted original replication region (*oriC*-*mioC*).

**Table S2.** Oligonucleotides used for genetic manipulation of *E. coli*.

| Primers used for genetic manipulation of *E. coli* | | | |
|---|---|---|---|
| Target | Target size | Position | Primer sequence (5'-3') |
| *oriC-mioC* | 801 bp | Forward | ATGGATTGAAGCCCGGGCCGTGGATTCTACTCA |
| | | Reverse | ATATCTAGAATGGCAGATATCACTCTTATCAGCGGCAGCACC |
| FRT-*kan*-FRT | 1495 bp | Forward | ATAGCTAGCGTGTAGGCTGGAGCTGCTTC |
| | | Reverse | ATAGCTAGCCATATGAATATCCTCCTTAG |
| *lacZ* | 1966 bp | Forward | GCAGACGATGGTGCAGGATATCCTGCTGATGAAGCAGAACAACTTTAACGCCGTGCGCATGGATTGAAGCCCGGGCCGTGGATTCTAC |
| | | Reverse | TTATTTTTGACACCAGACCAACTGGTAATGGTAGCGACCGGCGCTCAGCTGGAATTCCGCATATGAATATCCTCCTTAG |
| *dadZ-cvrA* | 385 bp | Forward | TTGTTGTAAGCCGGATCGGAGGCAACGTCTTCTGGGTGCAAAAAAATCATATGGATTGAAGCCCGGGCCGTGGATTCTAC |
| | | Reverse | AATTTAATGAAGCATCTATCGCCGGTTGCGATGCTTTGCTGAACGCAGCCTGATATGAATATCCTCCTTAG |
| Primers used for marker frequency analysis | | | |
| Name | Target gene | Position | Primer sequence (5′-3′) |
| *oriC* | *oriC* | Forward | GATCCCAGCTTATACGGTCC |
| | | Reverse | ATTAGGATCGCACTGCCCTG |
| *oriC*_R | *asnC* | Forward | GAAGCAGGCGGGGATCATTA |
| | | Reverse | GGGTAGTCTTTGGCGCTCTT |
| *oriC*_L | *mnmG* | Forward | GTTTATGGGCAATGCGTCCC |
| | | Reverse | CGAGGTTACTGCGGATCACA |
| *terC* | *uxaB - dws* | Forward | CACCGTTACTTCTGAACCCG |
| | | Reverse | ACAACATTAGCAGGCGCTGC |
| R1 | *rplI - ups* | Forward | CAAGTTCTGCCGTTTCACCG |
| | | Reverse | GACGCTGGTATTTTGCACGG |
| R2 | *mhpB* | Forward | GGACGTTTCACCAGCACTCT |

| | | | |
|---|---|---|---|
| | | Reverse | AATCTTTCCCGCTCCCCAAC |
| R3 | *pgl* | Forward | ATGTTGGTGTTCGCCCTGAG |
| | | Reverse | AAGACAAACTGCCCCTGGTG |
| R4 | *dhaR* | Forward | ATGGCGTGATTAGCTGGGAC |
| | | Reverse | AGTTCAGTGATTGCCCGTCC |
| L1 | *yhdY - dws* | Forward | GGTTGGGTATGTCGACGGAA |
| | | Reverse | GTACGCCCGGTGTTAAAACG |
| L2 | *gudX* | Forward | CAGGCCGCCGATTTTGATAC |
| | | Reverse | TTCAGCGCCTTACCTAGCAG |
| L3 | *hisM* | Forward | TTGTCGGTAGTGATAGGCGG |
| | | Reverse | CTGAACATACAGCGGCGTAC |
| L4 | *exoX* | Forward | AATCGTCAACCCCATGAGCC |
| | | Reverse | ATCCACGGTTTATCGGCGAC |

**Table S3.** Specific growth rates of *E. coli* MG1655 and O3 *lacZ dadX* under different growth conditions.

| Strain | LB medium | LB medium [a] | YT medium | TB medium | M9 medium [a] | M9 medium [b] |
|---|---|---|---|---|---|---|
| MG1655 | 1.614 ± 0.037 | 1.651 ± 0.021 | 1.927 ± 0.020 | 1.849 ± 0.023 | 0.598 ± 0.012 | 0.465 ± 0.011 |
| O3 *lacZ dadX* | 1.657 ± 0.010 | 1.663 ± 0.002 | 1.926 ± 0.034 | 1.815 ± 0.060 | 0.656 ± 0.010 | 0.464 ± 0.014 |

Data are presented as mean ± standard error.

[a] LB or M9 minimal medium supplemented with 0.4% glucose.

[b] M9 minimal medium supplemented with 0.2% glycerol.

**Table S4.** Reproducibility analysis of the enhanced growth rate of *E. coli* O3 *lacZ dadX*.

| Strain | Growth rate ($h^{-1}$) | Generation time (min) |
|---|---|---|
| MG1655 | 0.642 ± 0.020 | 64.851 ± 1.967 |
| O3 *lacZ dadX* | 0.739 ± 0.010 | 56.325 ± 0.736 |

Data are presented as mean ± standard error. Growth rate and generation time were determined using a micro-plate spectrophotometer.

**Table S5.** Growth condition-dependent changes in the cell size of *E. coli* derivatives cultured in LB or M9 medium supplemented with 0.4% glucose.

| Medium | Strain | Exponential phase [a] | | | Stationary phase [b] | | |
|---|---|---|---|---|---|---|---|
| | | Length (μm) | Diameter (μm) | Volume (μm$^3$) | Length (μm) | Diameter (μm) | Volume (μm$^3$) |
| M9 supplemented with 0.4% glucose | WT MG1655 | 2.723 ± 0.027 | 1.016 ± 0.005 | 1.930 ± 0.025 | 2.770 ± 0.038 | 1.010 ± 0.006 | 1.952 ± 0.036 |
| | O2 *lacZ* | 2.676 ± 0.029 | 0.954 ± 0.005 | 1.680 ± 0.022 | 2.803 ± 0.035 | 1.064 ± 0.006 | 2.184 ± 0.038 |
| | O3 *lacZ dadX* | 2.681 ± 0.030 | 1.024 ± 0.005 | 1.916 ± 0.024 | 2.765 ± 0.037 | 0.985 ± 0.006 | 1.866 ± 0.034 |
| | O2’ *lacZ dadX* | 2.964 ± 0.040 | 0.986 ± 0.005 | 2.009 ± 0.033 | 3.167 ± 0.047 | 1.168 ± 0.006 | 2.974 ± 0.054 |
| LB | WT MG1655 | 3.539 ± 0.049 | 1.163 ± 0.005 | 3.346 ± 0.058 | 2.388 ± 0.025 | 1.112 ± 0.006 | 1.950 ± 0.026 |
| | O2 *lacZ* | 3.574 ± 0.056 | 1.130 ± 0.007 | 3.241 ± 0.074 | 2.212 ± 0.024 | 1.115 ± 0.006 | 1.796 ± 0.028 |
| | O3 *lacZ dadX* | 3.649 ± 0.059 | 1.076 ± 0.004 | 2.988 ± 0.057 | 2.240 ± 0.025 | 1.166 ± 0.007 | 1.965 ± 0.028 |
| | O2’ *lacZ dadX* | 4.777 ± 0.164 | 1.120 ± 0.007 | 4.392 ± 0.184 | 2.880 ± 0.060 | 0.998 ± 0.006 | 1.954 ± 0.040 |

Data are presented as mean ± standard error.

[a] Cell length and diameter for each *E. coli* derivative was calculated after cultivation for 1 h in lysogeny broth (LB) or for 2 h in M9 minimal medium supplemented with 0.4% glucose.

[b] Cell length and diameter for each *E. coli* derivative was calculated after cultivation for 20 h in LB medium or 20 h in M9 minimal medium supplemented with 0.4% glucose.

**Table S6.** Summary of the flow cytometry analysis of *E. coli* cultured in LB medium.

| Strain | SGR [a] | G [b] | F4 [c] | F8 [d] | oriC/cell [e] | C+D period [f] | ai [g] | ai [h] |
|---|---|---|---|---|---|---|---|---|
| WT MG1655 | 1.720 ± 0.004 | 24.176 ± 0.049 | 0.487 ± 0.003 | 0.513 ± 0.003 | 6.051 ± 0.013 | 62.788 ± 0.138 | 0.403 ± 0.003 | 9.741 ± 0.079 |
| O2 *lacZ* | 1.723 ± 0.002 | 24.140 ± 0.024 | 0.436 ± 0.010 | 0.564 ± 0.010 | 6.257 ± 0.041 | 63.862 ± 0.294 | 0.355 ± 0.010 | 8.557 ± 0.223 |
| O3 *lacZ dadX* | 1.720 ± 0.001 | 24.176 ± 0.014 | 0.346 ± 0.003 | 0.654 ± 0.003 | 6.616 ± 0.012 | 65.902 ± 0.099 | 0.274 ± 0.003 | 6.625 ± 0.061 |

Data were presented as mean ± standard error.

[a] Specific growth rate ($h^{-1}$).

[b] Generation time (min).

[c] Fraction of cells with four chromosomes.

[d] Fraction of cells with eight chromosomes.

[e] Average number of *oriC* per cell.

[f] The time to complete both replication and cell division (min).

[g] Initial age of cell (0<ai<1).

[h] Initial age of cell (min).

# Supplementary Figures

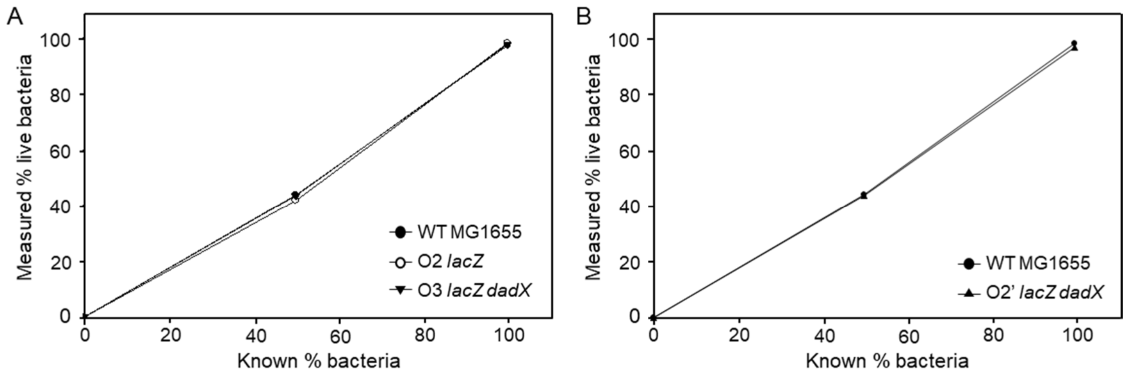


**Fig S1. Relative cell viability of strains with multiple or ectopic origins in lysogeny broth.** Bacterial cell viability was measured at the late log phase using flow cytometry. Cell viability of the strain was compared with that of wild-type MG1655. (A) Cell viability of O2 *lacZ* and O3 *lacZ dadX* with multiple replication origins. (B) Cell viability of O2' *lacZ dadX* with ectopic replication origins.

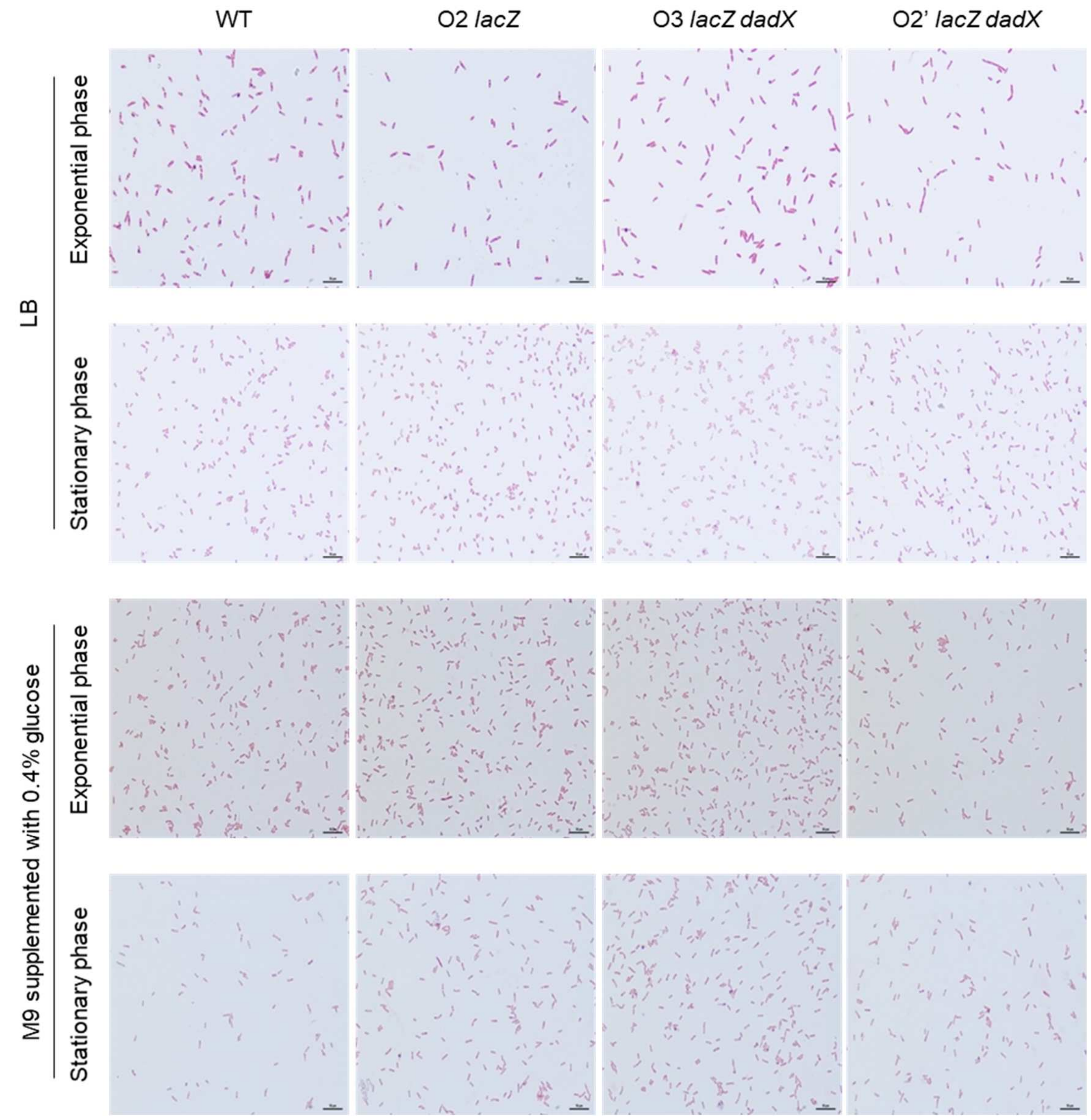


**Fig S2. Growth-dependent changes in the cell morphology of *Escherichia coli* strains.** The morphology of *E. coli* strains cultivated in LB or M9 medium with glucose was examined using Gram staining. Scale bar: 10 μm.

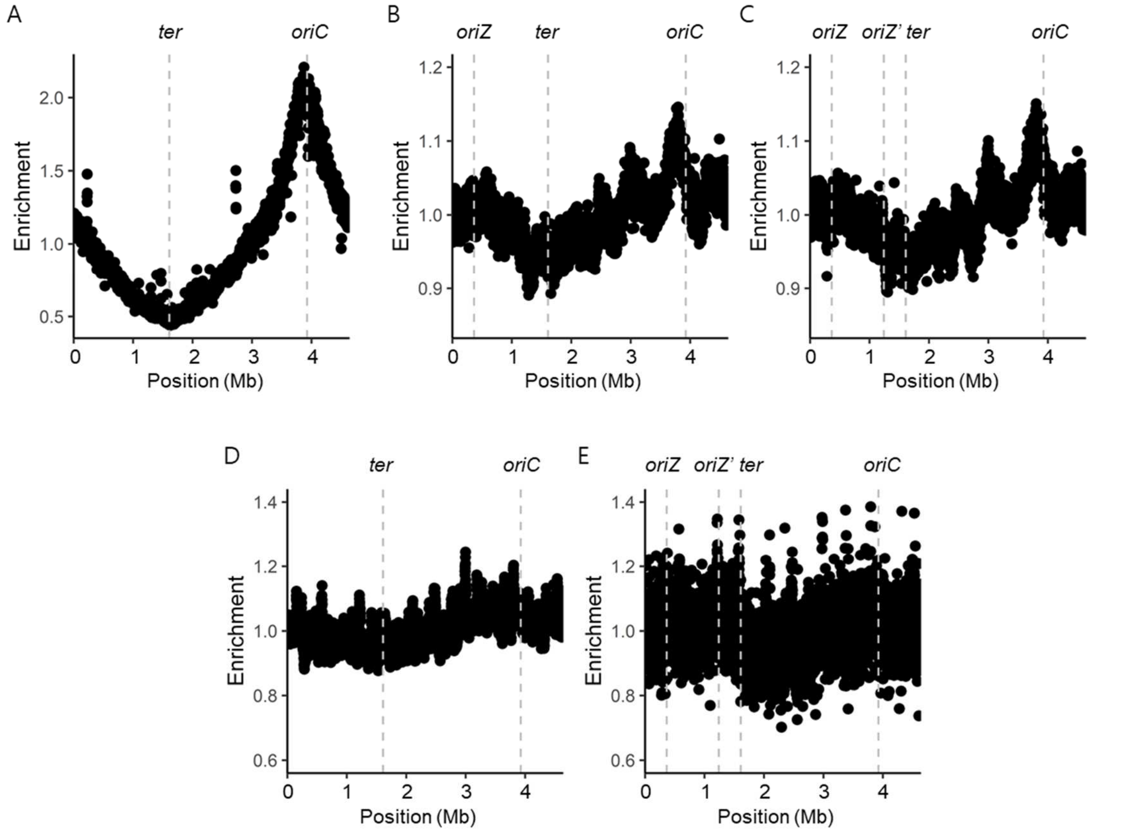


**Fig S3. Replication profiles of *Escherichia coli* strains.** Replication profiles of (A) wild-type MG1655 in LB, (B) O2 *lacZ* in LB, (C) O3 *lacZ dadX* in LB, (D) MG1655 in M9 medium with glucose, (E) O3 *lacZ dadX* in M9 medium with glucose. Genomic DNA from the cultures of MG1655, O2 *lacZ*, or O3 *lacZ dadX* was extracted and subjected to next-generation sequencing as described in the Materials and Methods section. The average coverage for every 1,000 bp in replicating sample DNA was normalized to reference DNA and plotted against each genomic position. The original replication origin (*oriC*), *oriZ*, *oriZ'*, and *ter* are shown in dotted lines.
